# Influence of Traumatic Brain Injury on Bone Healing Rate in Mandibular Fractures: A Prospective Comparative Study Using Ultrasonographic Assessment

**DOI:** 10.3390/cmtr19030031

**Published:** 2026-07-02

**Authors:** Kannan Balaraman, Vimal Kumar Kummari, S. Raja Sabapathy

**Affiliations:** 1 Department of Plastic, Hand, Maxillofacial and Burns Surgery, Ganga Hospital, Coimbatore 641043, India; 2 Department of Plastic and Reconstructive Surgery, MNJ Hospital, Hyderabad 500004, India; drvimalk@gmail.com

**Keywords:** traumatic brain injury, mandibular fractures, bone healing, ultrasonography, callus formation

## Abstract

Background: Accelerated bone healing in patients with traumatic brain injury (TBI) is well documented in long bone fractures but remains poorly studied in mandibular fractures. This study compared mandibular fracture healing rates in patients with and without TBI using high-frequency ultrasonography. Methods: A prospective comparative study was conducted from June 2020 to November 2021 at a single tertiary care center. All patients with mandibular fractures were enrolled in the study. They were divided into two groups as Group 1—with TBI—and Group 2—without TBI. All patients underwent either open reduction and internal fixation or intermaxillary fixation. Fracture healing was assessed weekly for four weeks using high-frequency ultrasonography (6–15 MHz) to evaluate callus formation patterns. Results: A total of 77 patients were enrolled in the study, of which 22 were in Group 1 and 55 in Group 2. Groups were comparable for age (33.23 ± 13.48 vs. 35.80 ± 13.65 years, *p* = 0.391) and gender distribution (*p* = 0.977). Mean time to initial callus formation was significantly shorter in Group 1 (15.45 ± 1.96 days) compared to Group 2 (19.98 ± 3.04 days, *p* < 0.001). By the second week, soft callus was evident in 27.3% of TBI patients versus 9.1% without TBI (*p* = 0.007). By the fourth week, 72.7% of Group 1 showed hard callus formation compared to 27.3% in Group 2 (*p* < 0.001). Glasgow Coma Scale scores showed significant inverse correlation with callus formation timing (*p* < 0.001). Conclusions: Mandibular fractures demonstrate accelerated healing in patients with TBI, with callus formation occurring approximately 4.5 days earlier. Ultrasonography provides an effective, radiation-free method for serial fracture assessment. These findings may inform surgical timing and follow-up protocols in polytrauma patients.

## 1. Introduction

Mandibular fractures represent 15.5–59% of all maxillofacial injuries, with road traffic accidents being the predominant etiology [1]. These fractures frequently occur in the context of polytrauma, with traumatic brain injury (TBI) present in 22–67% of cases [2,3]. Optimal fracture healing is essential for restoring masticatory function, phonation, and facial aesthetics.

The phenomenon of accelerated bone healing in patients with TBI has been extensively documented in long bone fractures [4,5,6]. Multiple mechanisms have been proposed, including release of osteogenic factors (bone morphogenic proteins, transforming growth factor-beta), neuropeptides (calcitonin gene-related peptide, substance P), and alterations in inflammatory mediators [7,8,9]. These factors are believed to be released from injured brain tissue and reach fracture sites through the compromised blood–brain barrier, creating a systemically enhanced osteogenic environment.

Despite substantial evidence in appendicular skeleton fractures, only one study to date has examined this phenomenon in mandibular fractures [10]. That study utilized computed tomography (CT) for assessment, raising concerns about cumulative radiation exposure during serial follow-up. High-frequency ultrasonography has emerged as a viable alternative for fracture assessment, offering real-time visualization without ionizing radiation [11,12].

This study aimed to compare mandibular fracture healing rates in patients with and without TBI using ultrasonographic evaluation, and to determine the timeline of callus formation in both populations.

## 2. Materials and Methods

### 2.1. Study Design and Setting

This prospective comparative study was conducted at a tertiary care center from June 2020 to November 2021. The study received approval from the Institutional Ethics Committee and Review Board. All participants provided written informed consent prior to enrollment.

### 2.2. Study Population

#### 2.2.1. Inclusion Criteria

- All patients presenting with mandibular fractures;
- TBI defined as contusion, hemorrhage (extradural, subarachnoid, intracerebral), or depressed skull fractures on CT imaging;
- All age groups;
- Treatment with either intermaxillary fixation (IMF) or open reduction and internal fixation (ORIF).

#### 2.2.2. Exclusion Criteria

- Inability to attend follow-up appointments;
- Current use of corticosteroids or immunosuppressive medications;
- Metabolic bone diseases;

Patients were divided into two groups based on presence (Group 1) or absence (Group 2) of TBI on initial CT brain imaging (Supplementary File S1).

#### 2.2.3. Clinical Assessment

All patients underwent comprehensive evaluation including the following:

- Clinical examination documenting facial symmetry, occlusion, dentition, and mouth opening;
- Neurological assessment by a neurosurgeon with Glasgow Coma Scale (GCS) scoring;
- CT imaging of face and brain;
- Documentation of associated injuries.

#### 2.2.4. Surgical Management

All patients were operated on by the same senior surgeon. Surgical approaches included the following:

- IMF for minimally displaced fractures;
- ORIF with 2 mm titanium plates and screws for displaced fractures;
- Plate selection (miniplates, maxiplates, or reconstruction plates) based on fracture configuration.

#### 2.2.5. Ultrasonographic Assessment

Serial ultrasonographic evaluation was performed by a single radiologist using a GE XD clear machine with a linear array transducer (6–15 Hz frequency). The first examination, conducted within one week post-operatively, served as the baseline. Subsequent evaluations were performed weekly for four weeks.

#### 2.2.6. Ultrasonographic Criteria

- **No callus:** Break in cortical continuity with no bridging tissue.
- **Soft callus:** Hyperechoic tissue bridging fracture site without calcification.
- **Soft and hard callus:** Mixed pattern with hyperechoic soft tissue and areas of calcification.
- **Hard callus:** Predominantly calcified bridging tissue.

### 2.3. Statistical Analysis

Statistical analyses were performed using SPSS version 16.0. Descriptive statistics included means and standard deviations for continuous variables, and frequencies and percentages for categorical variables. Independent sample *t*-tests compared continuous variables between groups. Chi-square tests evaluated categorical variables. Simple and multiple linear regression assessed relationships between variables and callus formation. Statistical significance was set at *p* < 0.05.

#### Sample Size

The sample size represented all patients meeting inclusion criteria during the study period. This was the first study using sequential ultrasonographic assessment of mandibular fractures, precluding formal sample size calculation. The COVID-19 pandemic additionally impacted patient volume during the study period.

## 3. Results

### 3.1. Patient Demographics

Seventy-seven patients were enrolled: 22 in Group 1 (28.6%) and 55 in Group 2 (71.4%). The study population was predominantly male (87%, *n* = 67), with a mean age of 35.0 ± 13.5 years (range 19–70 years).

Group 1 comprised 18 males (81.8%) and four females (18.2%), with a mean age of 33.23 ± 13.48 years. Group 2 included 49 males (89.1%) and six females (10.9%), with a mean age of 35.80 ± 13.65 years. Groups were statistically comparable for both age (*p* = 0.391) and gender distribution (*p* = 0.977) (Table 1).

### 3.2. TBI Characteristics

In Group 1, the mean GCS score was 12.59 ± 3.90 (range 3–15). TBI patterns included subdural hemorrhage (*n* = 4), extradural hemorrhage (*n* = 5), contusions (*n* = 8), and combinations thereof (*n* = 5).

### 3.3. Fracture Patterns and Management

Unilateral mandibular fractures occurred in 13 Group 1 patients (59.1%) and 21 Group 2 patients (38.2%). Bilateral fractures were present in nine Group 1 patients (40.9%) and 34 Group 2 patients (61.8%) (*p* = 0.095). Common fracture sites included parasymphysis (44.2%), angle (20.8%), body (18.2%), and condyle (16.8%).

IMF was performed in 11 Group 1 patients (50.0%) and 30 Group 2 patients (54.5%) (*p* = 0.718). Mean injury-to-surgery time was 38.27 h (median 21.50, IQR 16.50–48.00) for Group 1 and 50.53 h (median 46.00, IQR 20.00–72.00) for Group 2 (*p* > 0.05).

### 3.4. Callus Formation Timeline

**First-Week Assessment:** No callus formation was observed in either group during the first post-operative week, establishing a baseline for subsequent comparisons.

**Figure 1:**
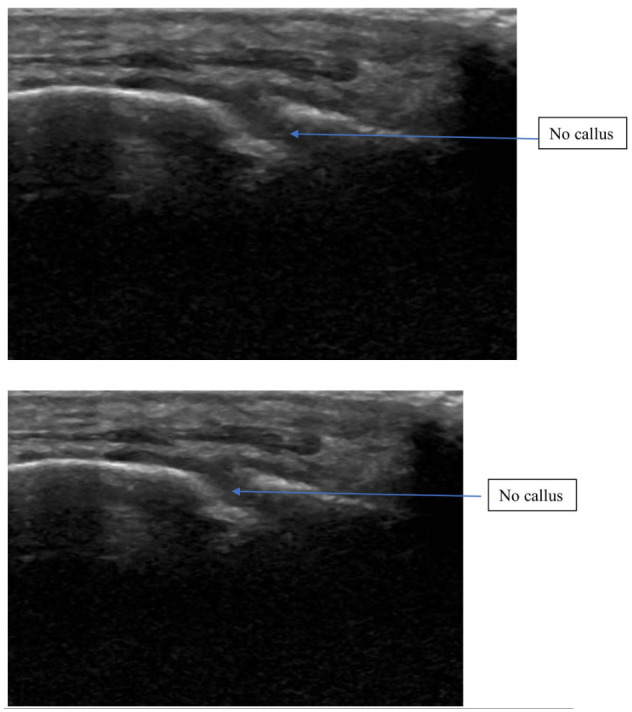
USG showing no callus at the fracture site (break in the continuity of white line).

**Earliest Callus Formation:** The earliest evidence of callus appeared at 12 days in Group 1 and 14 days in Group 2. Mean time to initial callus formation was 15.45 ± 1.96 days in Group 1 compared to 19.98 ± 3.04 days in Group 2 (*p* < 0.001), representing a 4.53-day difference (Figure 1).

**Second-Week Assessment:** In Group 1, 14 patients (63.6%) showed no callus, six (27.3%) demonstrated soft callus, and two (9.1%) showed both soft and hard callus. In Group 2, 50 patients (90.9%) had no callus while five (9.1%) showed soft callus (*p* = 0.007) (Table 2).

**Third-Week Assessment:** By the third week, all Group 1 patients demonstrated some degree of callus formation: eight (36.4%) had soft callus, 12 (54.5%) had mixed soft and hard callus, and two (9.1%) had hard callus. In contrast, 29 Group 2 patients (52.7%) still showed no callus, 22 (40.0%) had soft callus, and only four (7.3%) demonstrated mixed callus (*p* < 0.001).

**Figure 2:**
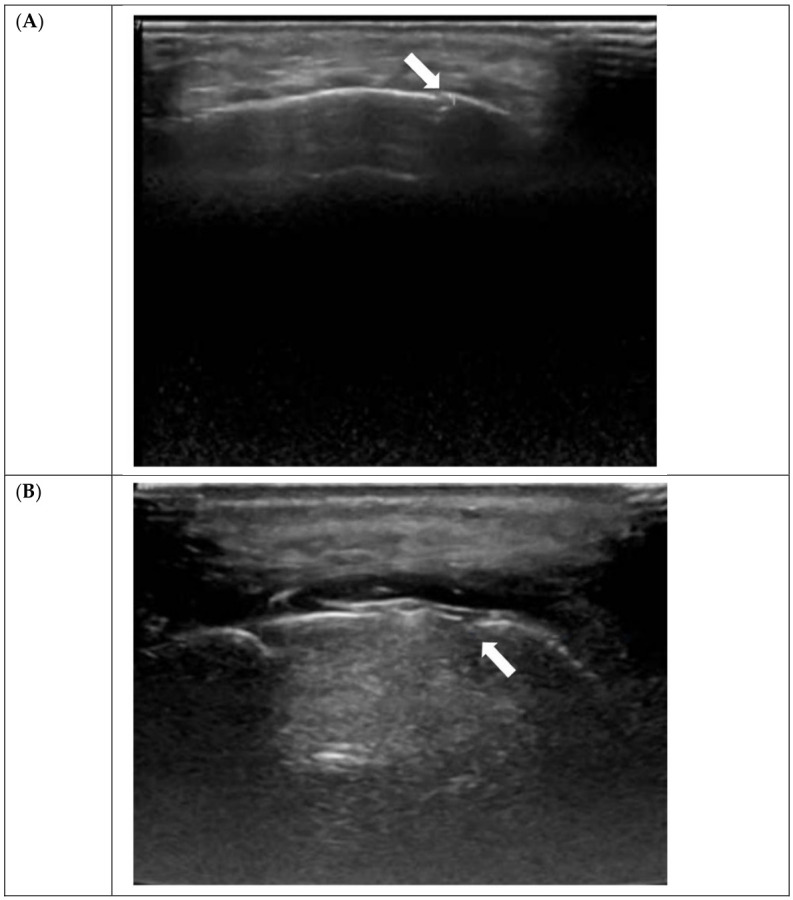
(**A**) Imaging of fracture was done post fixation in the third week showing inner hard callus (calcification) and outer soft callus (hyperechogenic) indicated by the arrow. (**B**) Imaging of fracture done in the fourth week post fixation showing hard callus (calcifications).

**Fourth-Week Assessment:** At four weeks, all Group 1 patients had progressed beyond isolated soft callus: six (27.3%) showed mixed callus and 16 (72.7%) had hard callus. In Group 2, 30 patients (54.5%) remained at the soft callus stage, 10 (18.2%) showed mixed callus, and only 15 (27.3%) had progressed to hard callus (*p* < 0.001) (Table 2, Figure 2).

### 3.5. Correlation with TBI Severity

Glasgow Coma Scale scores showed significant inverse correlation with callus formation timing (*p* < 0.001). Patients with lower GCS scores (indicating more severe TBI) consistently demonstrated earlier soft callus formation, typically by the second week.

### 3.6. Associated Injuries

Associated injuries showed variable distribution between groups. Upper limb injuries were significantly more common in Group 1 (40.9%) compared to Group 2 (5.5%) (*p* < 0.001). Chest injuries (13.6% vs. 7.3%, *p* = 0.380) and lower limb injuries (13.6% vs. 20.0%, *p* = 0.513) showed no significant differences between groups. These associated injuries did not significantly impact fracture healing rates.

## 4. Discussion

This study provides the first prospective evidence that mandibular fractures, like long bone fractures, demonstrate accelerated healing in the presence of TBI. Our findings reveal that callus formation occurs approximately 4.5 days earlier in TBI patients, with more rapid progression through healing stages.

### 4.1. Comparison with Existing Literature

Our findings align with the sole previous study on this topic by Huang et al., who reported earliest callus formation at 22 days in TBI patients versus 32 days in controls using CT assessment [10]. Our ultrasonographic approach detected callus formation earlier (12 vs. 14 days), likely reflecting superior soft tissue resolution and the ability to visualize pre-calcified callus stages.

In long bone fractures, multiple studies have documented similar phenomena. Spencer et al. demonstrated both enhanced healing response and shortened union time in patients with head injury [4]. Perkins and Skirving found earlier radiological union in femoral shaft fractures with associated TBI [5]. Our mandibular fracture data extend these findings to the craniofacial skeleton.

### 4.2. Pathophysiological Mechanisms

The accelerated healing observed likely results from multiple interconnected mechanisms:

**Osteogenic Factors:** Following TBI, elevated levels of bone morphogenic proteins (BMP-2, BMP-4), transforming growth factor-beta 1 (TGF-β1), and vascular endothelial growth factor (VEGF) create a systemically enhanced osteogenic environment [7,13]. These factors promote mesenchymal stem cell differentiation toward osteoblastic lineages and enhance angiogenesis at fracture sites.

**Neuropeptides:** Calcitonin gene-related peptide (CGRP), released from injured neural tissue, has been shown to improve local blood supply and accelerate fracture healing [14]. Song et al. demonstrated significantly elevated serum CGRP levels in TBI patients with enhanced fracture healing [15].

**Inflammatory Mediators:** Interleukin-6 (IL-6) and other cytokines released following TBI may promote osteoblast differentiation and activity [16]. Arachidonic acid, elevated after TBI, positively regulates bone gamma-carboxyglutamate protein expression and osteoblast proliferation [17].

**Blood–Brain Barrier Disruption:** TBI-induced increased permeability allows osteogenic macromolecules from the central nervous system to enter systemic circulation, reaching distant fracture sites [18].

**Metabolic Changes:** Mechanical ventilation and altered acid–base balance in TBI patients may create a more alkaline environment, facilitating calcium salt precipitation and hastening bone union [19].

**Correlation with TBI Severity:** Our finding of a significant correlation between GCS scores and healing rates suggests a dose–response relationship. Patients with more severe TBI (lower GCS) demonstrated earlier callus formation, supporting the hypothesis that greater neural injury releases higher concentrations of osteogenic factors. This observation has potential clinical implications for predicting healing trajectories in polytrauma patients.

**Ultrasonographic Assessment:** This study demonstrates the utility of high-frequency ultrasonography for serial mandibular fracture assessment. Traditional radiographic methods often fail to detect early soft callus formation, as calcification must reach sufficient density for radiographic visibility [20]. CT scanning, while offering superior spatial resolution, raises concerns about cumulative radiation exposure during serial follow-up.

Ultrasonography offers several advantages:

- Real-time visualization of soft tissue callus before calcification;
- Zero ionizing radiation exposure;
- Portability for bedside assessment;
- Cost-effectiveness compared to CT;
- Repeatability without safety concerns.

Previous studies have validated ultrasonography for long bone fracture healing assessment [11,12]. Our study extends these applications to mandibular fractures, demonstrating feasibility and diagnostic accuracy. The ability to differentiate soft callus, mixed callus, and hard callus provides detailed temporal characterization of the healing process.

Limitations include operator dependence and the inability to assess deep cortical surfaces due to the U-shaped mandibular anatomy. However, these limitations are offset by the advantages for serial monitoring in clinical practice.

**Clinical Implications:** Understanding accelerated healing in TBI patients has several practical applications:

**Surgical Timing:** Early definitive fixation may be particularly important in TBI patients to capitalize on the enhanced osteogenic environment while ensuring anatomically correct alignment before rapid callus consolidation.

**Follow-up Protocols:** TBI patients may require shorter intervals between initial follow-up visits to assess for premature or excessive callus formation, though our study did not identify cases of heterotopic ossification.

**Prognostic Information:** Knowledge of expected healing timelines can inform discussions with patients and families regarding functional recovery expectations.

**Rehabilitation Planning:** Earlier callus formation may allow accelerated progression to function-based rehabilitation, including dietary advancement and speech therapy.

### 4.3. Study Strengths and Limitations

**Strengths:**

- Prospective design with standardized protocols;
- Single-surgeon approach, minimizing technical variability;
- Single-radiologist assessment, ensuring consistency;
- Novel use of ultrasonography for serial assessment;
- Weekly follow-up, providing detailed temporal resolution.

**Limitations:**

- Unequal group sizes, though statistical methods addressed this disparity;
- Single-center study, potentially limiting generalizability;
- Sample size limited by study period and COVID-19 pandemic impact;
- Lack of biomechanical testing to correlate radiological with mechanical healing;
- Inability to quantify absolute callus volume with ultrasonography;
- Follow-up limited to four weeks, not extending to complete remodeling.

Future multicenter studies with larger sample sizes and longer follow-up periods would strengthen these findings. The investigation of specific biomarkers (BMP levels, CGRP concentrations) could provide mechanistic insights. Biomechanical testing could correlate ultrasonographic findings with functional healing strength.

**Unanswered Questions:**

Several questions merit further investigation:

- Does the quality of healing differ between TBI and non-TBI patients despite similar radiological appearance?
- What is the optimal surgical timing to maximize the osteogenic benefits while ensuring proper reduction?
- Do specific TBI types (contusion vs. hemorrhage) differentially affect healing rates?
- Does the enhanced healing persist beyond the initial four weeks into the remodeling phase?
- Are there long-term functional differences in mastication, jaw mobility, or pain between groups?

## 5. Conclusions

Mandibular fractures in patients with traumatic brain injury demonstrate significantly accelerated healing compared to isolated fractures, with callus formation occurring approximately 4.5 days earlier. By four weeks, 72.7% of TBI patients showed hard callus formation compared to only 27.3% without TBI. This phenomenon mirrors observations in long bone fractures and likely results from systemically elevated osteogenic factors, neuropeptides, and inflammatory mediators released following brain injury.

High-frequency ultrasonography provides an effective, radiation-free method for serial fracture assessment, offering advantages over traditional CT imaging for repeated follow-up examinations. The technique allows the visualization of soft callus before calcification and provides detailed temporal characterization of healing progression.

These findings have important clinical implications for surgical timing, follow-up protocols, and rehabilitation planning in polytrauma patients. Early definitive fixation may be particularly important in TBI patients to ensure anatomically correct healing before rapid callus consolidation. Clinicians should consider the accelerated healing timeline when planning follow-up intervals and progression to functional rehabilitation.

Further research with larger sample sizes, longer follow-up periods, and biomechanical correlation is warranted to fully characterize this phenomenon and optimize clinical management strategies for this challenging patient population.

## Figures and Tables

**Table 1:** Baseline demographic characteristics.

Parameter	Group 1 (TBI)	Group 2 (No TBI)	*p*-Value
Age (years), mean ± SD	33.23 ± 13.48	35.80 ± 13.65	0.391
Male gender, *n* (%)	18 (81.8%)	49 (89.1%)	0.977
Female gender, *n* (%)	4 (18.2%)	6 (10.9%)	-

**Table 2:** Callus formation patterns by week.

Assessment	Group 1 (*n* = 22)	Group 2 (*n* = 55)	*p*-Value
**Week 2**			0.007
No callus	14 (63.6%)	50 (90.9%)	
Soft callus	6 (27.3%)	5 (9.1%)	
Mixed callus	2 (9.1%)	0 (0%)	
**Week 3**			<0.001
No callus	0 (0%)	29 (52.7%)	
Soft callus	8 (36.4%)	22 (40.0%)	
Mixed callus	12 (54.5%)	4 (7.3%)	
Hard callus	2 (9.1%)	0 (0%)	
**Week 4**			<0.001
Soft callus	0 (0%)	30 (54.5%)	
Mixed callus	6 (27.3%)	10 (18.2%)	
Hard callus	16 (72.7%)	15 (27.3%)	

## Data Availability

The original contributions presented in this study are included in the article/Supplementary Material. Further inquiries can be directed to the corresponding authors.

## Supplementary Materials

The following supporting information can be downloaded at: https://www.mdpi.com/article/10.3390/cmtr19030031/s1. File S1: Master Chart (Group 1 and Group 2).
