# Supplementary material for: Influence of Traumatic Brain Injury on Bone Healing Rate in Mandibular Fractures: A Prospective Comparative Study Using Ultrasonographic Assessment
Source: Craniomaxillofac Trauma Reconstr. 2026 Jul 2;19(3):31. doi: 10.3390/cmtr19030031 (PMC13398041; doi:10.3390/cmtr19030031)
Supplement: Supplementary file 1 [file cmtr-19-00031-s001.zip › cmtr-4261835-supplementary.pdf]

MASTER CHART

GROUP 1

SL NO	ID NO	NAME	AGE (Years)	SEX	MODE OF INJURY	CLINICAL EXAMINATION					CT FACE FINDINGS		CT BRAIN	SITE OF MANDIBLE FRACTURE	ASSOCIATED INJURIES				INURY TO SURGERY TIME	PROCEDURE	TYPE OF FIXATION	POST OP IMF	USG			
						FACIAL SYMMETRY	OCCLUSION	TEETH	MOUTH OPENING	GCS TOTAL = 15	MANDIBLE	OTHERS			CHEST INJURY	ABDOMINAL INJURY	UPPER LIMB	LOWER LIMB		YES			1st usg (1st week)	2nd usg (2nd week)	3rd usg(3rd week)	4th usg(4th week)
1	1120094376	Mr.GOPALAKRISHNAN. N	30	Male	RTA (2 wheeler vs tractor)	present	Occlusion derranged.	normal	restricted	15	unilateral mandible	Unilateral zygoma fracture	SDH	left parasymphysis	NONE	NONE	NONE	NONE	30HRS	OPEN REDUCTION AND INTERNAL FIXATION - LEFT PARASYMPHYSIS	MAXIPLATE	NO	no callus	soft callus	soft and hard callus	Hard callus
2	1120094016	Mr.KARTHIK QURAV	19	Male	RTA (2 wheeler vs 2 wheeler)	present	Occlusion derranged.	normal	restricted	9	bilateral mandible	Bilateral maxilla fracture	EDH	right angle and parasymphysis of mandible	NONE	NONE	NONE	PRESENT	36HRS	OPEN REDUCTION AND INTERNAL FIXATION - RIGHT PARASYMPHSIS OF MANDIBLE AND ZYGOMATICOMAXILARY COMPLEX	MAXIPLATE	NO	no callus	soft callus	soft and hard callus	Hard callus
3	1120051268	Mr.ALAGUTHAMILVANANT	31	Male	RTA (2 wheeler skid and fall)	Flattening of cheek present over left side.	Occlusion derranged.	normal	restricted.	15	comminuted bilateral mandible	bilateral maxilla	LEFT TENTORIAL BLEED RIGHT TEMPORAL FRACTURE.	RIGHT PARASYMPHYSIS AND MINIMALLY DISPLACED LEFT SUBCONDYLE	NONE	NONE	NONE	NONE	18HRS	OPEN REDUCTION AND INTERNAL FIXATION - RIGHT PARASYMPHYSIS FRACTURE OF MANDIBLE WITH IMF AND LEFT ZYGOMA ELEVATION. DEBRIDEMENT, REALIGNMENT AND REPAIR OF RAW AREA WITH FULL THICKNESS SKIN LOSS BEHIND RIGHT EAR (POST AURICULAR REGION).	MINIPLATES	YES	no callus	no callus	soft callus	soft and hard callus
4	1120051497	Mr.MANIKANDAN. S	21	Male	RTA (two wheeler - self skid)	present	Deranged occlusion	normal	restriction	15	comminuted bilateral mandible		RIGHT CEREBELLAR SAH TENTORIAL BLEED RIGHT INTER HEMISPHERIC BLEED.	RIGHT CONDYLE AND PARASYMPHYSIS	NONE	NONE	NONE	NONE	19HRS	ORIF - RIGHT CONDYLE AND PARASYMPHYSIS FRACTURE OF MANDIBLE WITH IMF. MEDICAL MANAGEMENT OF HEAD INJURY.	MINIPLATES	YES	no callus	no callus	soft callus	Hard callus
5	1120050809	Ms.KAVIYAPPRIYA.Y	19	Female	(2 wheeler vs tractor)	present	Deranged occlusion	Avulsed Teeth 11, 22, 33, grade II mobility - 21	restriction	10	comminuted bilateral mandible	bilateral maxilla	PNEUMOCEPHALUS EDH SDH CONTUSION – RIGHT CEREBELLAR HEMISPHERE	PARASYMPHYSIS AND ANGLE (RIGHT)	PRESENT	NONE	PRESENT	NONE	63HRS	OPEN REDUCTION AND INTERNAL FIXATION - LEFT MANDIBULAR PARASYMPHYSIS AND RIGHT ANGLE FRACTURE. OPEN REDUCTION AND INTERNAL	LOCKING PLATE MINIPLATES	YES	no callus	soft callus	Hard callus	Hard callus

SL NO	ID NO	NAME	AGE (Years)	SEX	MODE OF INJURY	CLINICAL EXAMINATI ON					CT FACE FINDINGS		CT BRAIN	SITE OF MANDIBLE FRACTURE	ASSOCIATE D INJURIES				INURY TO SURGERY TIME	PROCEDUR E	TYPE OF FIXATION	POST OP IMF	USG			
																				FIXATION - LEFT ZYGOMATICO MAXILLARY COMPLEX FRACTURE.						
6	11200 60906	Mr.DHARMAL INGAM A	68	Male	RTA (pedestrian vs 2 wheeler)	present	Deranged occlusion	normal	restri ction	15	comminu ted bilateral mandible	bilateral maxilla	LEFT BASAL FRONTAL FRACTURE WITH PNEUMOCEP HALUS	RIGHT RAMUS AND ANGLE.	NONE	NONE	PRESE NT	NONE	120HR S	OPEN REDUCTION AND INTERNAL FIXATION OF ANGLE FRACTURE OF MANDIBLE	MINIPLATE	NO	no callus	no callus	soft and hard callus	Hard callus
7	11200 51123	Mr.SAKTHIVE L A	27	Male	RTA (2 wheeler vs 2 wheeler)	present	Maxilla mobility present with anterior open bite. - Palatal split present.	normal	restri ction	3	unilateral mandible	bilateral maxilla and frontal bone	SEVERE HEAD INJURY : DIFFUSE CEREBRAL EDEMA.	MANDIBU LAR PARASYM PHYSIS	PRESE NT	NONE	NONE	NONE	9HRS	OPEN REDUCTION AND INTERNAL FIXATION OF MANDIBULAR PARASYMPHYSIS, BILATERALMAXIL LA WITH IMF ELASTICS, CLOSED REDUCTION OF NASAL BONE. DEBRIDEMENT, REPAIR OF LACERATIONS OVER LEFT LATERAL SIDE OF EYE.	MAXIPLATE MINIPLATE	YES	no callus	no callus	hard callu and soft callus	Hard callus
8	11200 60070	Mr.AMMASAI APPAN	48	Male	History of fall from a 2 wheeler	present	Deranged occlusion	normal	restri ction	11	bilateral mandible	bilateral maxilla	EDH - right isde	RIGHT MANDIBU LAR BODY AND LEFT CONDYLE	NONE	NONE	NONE	NONE	1OHR S	ORIF - FRACTURE RIGHT MANDIBULAR BODY WITH INTERMAXILLARY FIXATION FOR CONDYLE FRACTURE - FRACTURE RIGHT ZYGOMA ELEVATION	MINIPLATES	YES	no callus	no callus	soft callus	Hard callus
9	11200 81335	Mr.CHIZEYAN .K	55	Male	RTA (2 wheeler vs 2 wheeler)	present	Deranged occlusion	normal	restri cted.	15	unilateral mandible	bilateral maxilla and frontal bone	CONTUSION - LEFT FRONTAL AND BASIFRONT AL LOBE. EXTRADURA L HEMORRHA GE COMMINUTE D FRACTURE - FRONTAL BONE	LEFT PARASYM PHYSIS OF MANDIBL E	NONE	NONE	PRESE NT	NONE	23HRS	WOUND EXPLORATION AND PRIMARY CORNEO SCLERAL TEAR REPAIR FOR LEFT EYE. PARTIAL EXCISION AND PARTIAL ELEVATION OF FRONTAL DEPRESSED FRACTURE RECONSTRUCTION OF FRONTAL BONE DEFECT WITH TITANIUM MESH, OPEN REDUCTION AND INTERNAL FIXATION OF LEFT PARASYMPHYSIS MANDIBLE AND LEFT MAXILLA AND MID PALATAL REGION,	MINIPLATES	NO	no callus	no callus	hard callu and soft callus	hard callus

SL NO	ID NO	NAME	AGE (Years)	SEX	MODE OF INJURY	CLINICAL EXAMINATION					CT FACE FINDINGS		CT BRAIN	SITE OF MANDIBLE FRACTURE	ASSOCIATED INJURIES				INURY TO SURGERY TIME	PROCEDURE	TYPE OF FIXATION	POST OP IMF	USG			
																				CLOSED REDUCTION OF NASAL BONE FRACTURE.						
10	1120084134	Mr.SURYAPRAKASH G	24	Male	two wheeler rider hit by a another two wheeler	present	Deranged occlusion	avulsed Teeth 11, 22, 33, grade II mobility - 21 avulsed tooth upper both central incisors, left lateral incisors, left lower cannine	restricted.	6	comminuted unilateral mandible	bilateral maxilla	HEMORRHAGIC CONTUSIONS IN BILATERAL FRONTAL LOBE. LEFTPARIETAL LOBE HYPODENSE CONTUSION	LEFT PARASYMPHYSIS, LEFT CONDYLAR PROCESS	NONE	NONE	NONE	NONE	4HRS	OPEN REDUCTION AND INTERNAL FIXATION OF LEFT PARASYMPHYSIS, LEFT FRONTOZYGOMATIC BUTTRESS, LEFT MAXILLA, INTERMAXILLARY FIXATION WITH SCREWS DEBRIDEMENT, REPAIR AND REALIGNMENT OF WOUND OVER UPPER & LOWER LIP, REMOVAL OF MOBILE 3	LOCKING PLATE MINIPLATES	YES	no callus	no callus	soft callus	hard callu and soft callus
11	1120102640	Mr.THANGARAJ.P	29	Male	RTA, (His two wheeler hit against the divider)	present	Deranged occlusion	Multiple missing lower anterior front teeth - Mobile upper anterior teeth.	restricted.	7	comminuted unilateral mandible	right maxilla, frontal bone	BILATERAL FRONTAL HAEMORRHAGIC CONTUSION DEPRESSED FRONTAL BONE FRACTURE	PARASYMPHYSIS	NONE	NONE	NONE	NONE	40HRS	PARTIAL EXCISION AND PARTIAL ELEVATION OF THE LEFT FRONTAL DEPRESSED FRACTURE COVERING OF THE BONY DEFECT WITH THE TITANIUM MESH. OPEN REDUCTION AND INTERNAL FIXATION LEFT PARASYMPHYSIS FRACTURE OFMANDIBLE WITH INTERMAXILLARY FIXATION WITH SCREWS DEBRIDEMENT, REALIGNMENT AND REPAIR DONE FOR LACERATIONS OVER FOREHEAD, TONGUE AND UPPER LIP.	RECON PLATE	NO	no callus	soft callus and hard callus	soft callus and hard callus	hard callus seen
12	1120102569	Mrs.SUGANTHI	30	Female	RTA (vans hit over tree)	present	Deranged occlusion	normal	restricted.	15	unilateral mandible	bilateral maxilla	FRACTURE OF RIGHT FRONTAL BONE EXTENDING INTO FRONTAL SINUS AND POSTERIORLY INTO THE ROOF OF ETHMOID SINUS, LEFT	LEFT PARASYMPHYSIS AND RIGHT ANGLE OF MANDIBLE.	PRESENT	NONE	PRESENT	PRESENT	20HRS	DEBRIDEMENT AND KNEE SPANNING AND TIBIA EXFIX LEFT SIDE OPEN REDUCTION AND INTERNAL FIXATION TBW LEFT PATELLA DEBRIDEMENT AND SUTURING WOUND OVER LEFT DISTAL	MINIPLATES	NO	no callus	no callus seen	soft callus and hard callus	hard callus

SL NO	ID NO	NAME	AGE (Years)	SEX	MODE OF INJURY	CLINICAL EXAMINATION					CT FACE FINDINGS		CT BRAIN	SITE OF MANDIBLE FRACTURE	ASSOCIATED INJURIES				INURY TO SURGERY TIME	PROCEDURE	TYPE OF FIXATION	POST OP IMF	USG			
													OPTIC CANAL AND LEFT MASTOID TEMPORAL BONE. HEMOSINUS AND LEFT HEMOMAST OID NOTED.							FEMUR AND BILATERAL FOREARM AND LEFT ARM.<br>OPEN REDUCTION AND INTERNAL FIXATION - FRACTURE BILATERAL MANDIBLE (LEFT PARASYMPHYSIS & RIGHT ANGLE OF MANDIBLE).<br>OPEN REDUCTION AND INTERNAL FIXATION WITH PLATING LEFT HUMERUS AND LEFT DISTAL 3RD OF RADIUS AND ULNA.<br>DEBRIDEMENT ,OPEN REDUCTION INTERNAL FIXATION OF LEFT DISTAL FEMUR WITH LOCKING COMPRESSION PLATING						
13	11201 14501	Mr.UTHAYKU MAR	25	MAL E	RTA 2 wheeler vs 2 wheeler	Flattened left side of face	Deranged occlusion(anterior open bite)	normal	restri cted.	15	bilateral mandible	left zygoma	LEFT TEMPORAL BONE FRACTURE WITH CONTUSION	LEFT PARASYM PHYSIS	NONE	NONE	NONE	NONE	18HRS	OPEN REDUCTION AND INTERNAL FIXATION OF LEFT PARASYMPHYSIS LEFT FRONTOZYGOMAT IC BUTTRESS LEFT ORBITAL FLOOR RECONSTRUCTION -NTERMAXILLARY FIXATION FOR BILATERAL CONDYLE FRACTURE<br>- DEBRIDEMENT, REPAIR OF LEFT EYE WOUND<br>- OPEN REDUCTION AND INTERNAL FIXATION RIGHT CONDYLAR FRACTURE OF MANIDIBLE	MAXIPLATE MINIPLATE	YES	no callus	no callus seen	soft callus and hard callus	hard callus
14	11210 10150	Mr.VENKATE SAN	27	MAL E	Alleged RTA (Fell down from 2 wheeler)	present	derangem ent of occlusion	UPPER ANTERI OR TOOTH AVULSI ON	restri cted.	15	unilateral mandible	unilatera l zygoma fracture	EDH PNEUMOCEP HALUS INTRAVENT RICULAR HEMORRHA GE SKULL BASE FRACTURE	left parasymphy sis of mandible	NONE	NONE	PRESE NT	NONE	72HRS	OPEN REDUCTION AND INTERNAL FIXATION OF MANDIBLE, ZYGOMA, PALATAL SPLIT, LEFT FLOOR OF ORBIT	MINIPLATES	YES	no callus	soft callus	hard callus	hard callus
15	11210 35936	Mr.SIVAKUM AR C	23	MAL E	RTA (2 wheeler vs 4 wheeler)	present	deranged	normal	restri cted.	15	bilateral mandible		RIGHT FRONTAL AND ORBITAL FRACTURE	RIGHT ANGLE AND LEFT BODY OF MANDIBLE	NONE	NONE	PRESE NT	NONE	48HRS	OPEN REDUCTION AND INTERNAL FIXATION OF BILATERAL MANDIBLE	MINIPLATES	YES	No callus	No callus	Soft and hard callus	Hard callus

SL NO	ID NO	NAME	AGE (Years)	SEX	MODE OF INJURY	CLINICAL EXAMINATION					CT FACE FINDINGS		CT BRAIN	SITE OF MANDIBLE FRACTURE	ASSOCIATED INJURIES				INURY TO SURGERY TIME	PROCEDURE	TYPE OF FIXATION	POST OP IMF	USG			
16	11210 37739	Mr.NETHAJI	24	MALE	skid and fall from bike	present	deranged	normal	restricted.	15	unilateral mandible	unilateral zygoma fracture	FRONTAL BONE FRACTURE WITH PNEUMOCEPHALUS	right parasymphysis of mandible	NONE	NONE	PRESENT	NONE	48HRS	OPEN REDUCTION AND INTERNAL FIXATION - RIGHT PARASYMPHYSIS, RIGHT ZYGOMA ELEVATION	MINIPLATES	NO	no callus	No callus	Soft callus (pod 20)	Soft and hard callus
17	11210 92213	Ms.FASEEHA	24	Female	RTA (Car vs Echier van)	present	deranged	normal	restricted.	15	bilateral mandible		BILATERAL TEMPORAL BONE FRACTURE WITH EDH	right parasymphysis of mandible.	NONE	NONE	PRESENT	PRESENT	168HRS	OPEN REDUCTION AND INTERNAL FIXATION - RIGHT PARASYMPHYSIS,	MINIPLATE	NO	No callus	soft callus	Soft and hard callus	Hard callus
18	11210 61520	Mr.MANIKANDAN. S	46	Male	RTA (2 wheeler vs 2 wheeler)	present	deranged	normal	restricted.	6	comminuted unilateral mandible	unilateral zygoma	DIFFUSE AXONAL INJURY WITH LOBAR CONTUSION	right parasymphysis of mandible.	NONE	NONE	NONE	NONE	36HRS	OPEN REDUCTION AND INTERNAL FIXATION PLATING OF SEGMENAL FRACTURE OF MANDIBLE	RECON PLATE	NO	No callus	Soft callus	soft and hard callus	hard callus
19	11210 72590	Mr.RAMASAMY	45	Male	RTA(fell down from 2 wheeler)	present	deranged	MULTIPLE TEETH FRACTURE UPPER ANTERIOR	restricted.	15	Comminuted displaced fracture of body of mandible on left side and para symphys bilateral mandible is also involving alveolar arch.		TEMPORAL LOBE CONTUSION, TENTORIAL BLEED, SAH, EDH	FRACTURE OF LEFT BODY OF MANDIBLE.	NONE	NONE	NONE	NONE	12HRS	OPEN REDUCTION AND INTERNAL FIXATION LEFT BODY OF MANDIBLE	MAXIPLATE	NO	no callus	Soft callus	soft and hard callus	hard callus
20	11210 96139	Mr.SATHASIVAM N	46	Male	RTA (2 wheeler VS bus)	present	deranged	MULTIPLE TOOTH AVULSION	restricted.	15	unilateral mandible	bilateral maxilla	DEPRESSED COMMUNITED FRONTAL BONE FRACTURE WITH PNEUMOCEPHALUS	symphysis of mandible	NONE	NONE	NONE	NONE	18HRS	OPEN REDUCTION AND INTERNAL FIXATION OF SYMPHYSIS MANDIBLE	MINIPLATES	yes	no callus	no callus	Soft callus	soft and hard callus
21	11210 97820	Ms.VEDA SRUTI . M	22	Female	RTA (2 wheeler vs 4 wheeler)	present	deranged	MULTIPLE TOOTH AVULSION	restricted.	15	unilateral mandible	bilateral maxilla	RIGHT TEMPORAL BLEED, RIGHT TEMPORAL CONTUSION, SDH, CEREBRAL EDEMA	left parasymphysis of mandible	NONE	NONE	PRESENT	NONE	10HRS	OPEN REDUCTION AND INTERNAL FIXATION - LEFT PARASYMPHYSIS	MINIPLATES	yes	no callus	no callus	Soft callus	soft and hard callus
**22**	11201 02676	Mr.ANTHONI. C.A	48	M	bison	present	deranged	none	restricted.	15	unilateral mandible	nil	FRONTAL LOBE CONTUSION	RIGHT BODY OF MANDIBLE	nil	nil	nil	nil	30hrs	ORIF - right body of mandible	MAXIPLATE MINIPLATE	yes	no callus	No callus	Soft callus	soft and hard callus

GROUP 2

S.No	ID No	NAME	AGE (YEARS)	SEX	MODE OF INJURY	GCS	CT FACE FINDINGS		Tooth	SITE OF MANDIBLE FRACTURE	ASSOCIATED INJURIES				INJURY TO SURGERY TIME (hrs)	PROCEDURE	TYPE OF FIXATION	POST OP IMF	ULTRASONOGRAM			
							MANDIBLE	OTHERS			CHEST INJURY	ABDOMINAL INJURY	UPPER LIMB	LOWER LIMB					1st usg	2nd usg (2week)	3rd usg (3week)	4th usg (4week)
1	1120061463	Mr.RAJKUMAR	21	M	Fall	15	communited unilateral mandible	unilateral zygoma		right body	nil	nil	nil	nil	72	ORIF of body of mandible, Left ZMC,	recon plate	Yes	no call us	no callus	no callus	soft callus
2	1120090149	Mr.CHENNAIYAN.M	57	M	RTA	15	unilateral mandible	unilateral zygoma		RIGHT PARASYMPHYSIS OF MANDIBLE	nil	nil	nil	nil	46	ORIF OF R Parasymphysis elevation of zygoma	mid plate	No	no call us	soft callus	soft and hard callus	hard callus
3	1120092678	Mr.NANDHA KUMAR	38	M	RTA	15	unilateral mandible	unilateral zygoma		RIGHT BODY	nil	nil	nil	nil	60	ORIF Of body mandible, right zygoma elevation	mid plate	No	no call us	no callus	no callus	soft callus
4	1120101322	Mr.PARAMESHWARAN	56	M	RTA	15	communited biilateral mandible			bilateral parasymphysis	"nil "	nil	nil	nil	32	ORIF of bilateral parasymphysis	MAXIPLATE	Yes	no call us	no callus	no callus	soft callus
5	1120091154	Mr.ELANGOVAN K	24	M	"RTA "	15	bilateral mandible			left parasymphysis and angle	"nil "	"nil "	"nil "	nil "	37	ORIF of left parasymphysis	MAXIPLATE	No	no call us	soft callus seen	soft and hard callus	hard callus
6	1120090191	Mr.RAVI . M	35	M	RTA	15	bilateral mandible	bilateral maxilla	Avulsed 1 3 , 1 4	right body left condyle	nil	nil	Yes	Yes	72	ORIF R body and left condyle, right zygoma	MINIPLATE	Yes	no call us	no callus	no callus	soft callus
7	1120091115	Mr.SHANMUGA SUNDHARAM	60	M	RTA	15	bilateral mandible	bilateral maxilla	Multiple avulsed to other	parasymphysis and angle of mandible	nil	nil	nil	nil	105	ORIF left parasymphysis and ramus of mandible, elevation of nasomaxillary complex	MAXIPLATE	Yes	no call us	no callus	soft callus	soft callus
8	1120100116	Ms.SWASHIKA .R	20	F	Fight	15	bilateral mandible		None	right parasymphysis and left angle	nil	nil	nil	nil	106	ORIF right para and left angle	MAXIPLATE MINIPLATE	No	no call us	no callus	no callus	soft callus
9	1120102226	Mrs.VIJAYA	45	F	RTA	15	bilateral mandible		Mobile tooth 21 22	left condyle right parasymphysis	nil	nil	nil	nil	60	IMF	MINIPLATE	Yes	no call us	no callus	soft callus	soft and hard callus
10	1120092399	Mr.VINAYAGAMOORTHI .P	35	M	RTA	15	communited unilateral mandible	bilateral maxilla	Mobile 12	parasymphysis	nil	nil	nil	nil	12	ORIF Left parasymphysis, left frontozygomatic , nasal bone reduction	MAXIPLATE	Yes	no call us	no callus	soft callus	hard callus seen
11	1120030005	Mr.RAVICHANDRAN P	52	M	RTA	15	bilateral mandible			RIGHT CONDYLE AND PARASYMPHYSIS LEFT BODY OF MANDIBLE.	nil	nil	nil	nil	10	ORIF - right parasymphysis, left body, right condyle	MAXIPLATE	No	no call us	no callus	soft callus	hard callus

S.No	ID No	NAME	AGE (YEARS)	SEX	MODE OF INJURY	GCS	CT FACE FINDINGS		Tooth	SITE OF MANDIBLE FRACTURE	ASSOCIATED INJURIES				INJURY TO SURGERY TIME (hrs)	PROCEDURE	TYPE OF FIXATION	POST OP IMF	ULTRASONOGRAM			
12	1120052558	Mr.RAMKUMAR P	28	M	RTA	15	bilateral mandible		Fractured teeth 21 24	BILATERAL CONDYLE AND SYMPHYSIS	nil	nil	nil	nil	48	ORIF left condyle, symphysis	MINIPLATE	Yes	no call us	no callus	soft callus	hard callus
13	1120060741	Mr.NATARAJAN	55	M	Fall	15	bilateral mandible		None	RIGHT PARASYMPHYSIS AND LEFT CONDYLE	Yes	No	Yes	nil	60	ORIF right parasymphysis	MAXIPLATE	Yes	no call us	no callus	no callus	soft callus
14	1120060900	Mr.RATHINAVEL P	29	M	RTA	15	unilateral mandible		Loose lower incisors right side	SYMPHYSIS	nil	nil	nil	nil	78	ORIF symphysis	MAXIPLATE	No	no call us	no callus	no callus	soft callus
15	1120061320	Mr.SAKTHIVEL M	32	M	RTA	15	communited biilateral mandible			SYMPHYSIS	nil	nil	nil	nil	13	ORIF symphysis	MAXIPLATE	No	no call us	no callus	no callus	soft callus
16	1120051687	Mrs.AMUTHA	35	F	RTA	15	bilateral mandible		None	bilateral condyle and symphysis	nil	nil	nil	nil	44	ORIF symphysis	MAXIPLATE	Yes	no call us	no callus	no callus	soft callus
17	1120071707	Mr.DHAMODHARAN	60	M	RTA	15	bilateral mandible		None	right parasymphysis and bilateral condyle	nil	nil	nil	nil	28	ORIF right parasymphysis	MAXIPLATE	Yes	no call us	no callus	soft callus	soft and hard callus
18	1120061914	Mr.ELANGOVAN	27	M	RTA	15	bilateral mandible		None	right parasymphysis left condyle	nil	nil	nil	nil	20	ORIF right parasymphysis left subcondylar	MINIPLATE	Yes	no call us	soft callus	soft and hard callus	hard callus
19	1120062152	Mr.MURUGAVEL M	23	M	RTA	15	bilateral mandible		Fractured teeth 11 21	right condyle and left angle	nil	nil	nil	nil	90	ORIF right condlye, left angle	MINIPLATE	Yes	no call us	soft callus seen	soft plus hard callus	hard callus
20	1120062065	Mr.MUTHUKUMAR. R	29	M	RTA	15	bilateral mandible		None	right parasymphysis and left angle	nil	nil	nil	nil	72	ORIF left angle	MINIPLATE	Yes	no call us	no callus	soft callus	soft and hard callus
21	1120061464	Mr.SENTHILKUMAR N	35	M	RTA	15	communited biilateral mandible		None	RIGHT PARASYMPHYSIS AND LEFT BODY OF MANDIBLE FRACTURE AND LEFT CONDYLE OF MANDIBLE	nil	nil	nil	nil	48	ORIF - right parasymphysis, left body	MAXIPLATE MINIPLATE	Yes	no call us	no callus	no callus	soft callus
22	1120090198	Mr.SARAVANAN	27	M	Fall	15	bilateral mandible		None	RIGHT PARASYMPHYSIS AND LEFT CONDYLE OF MANDIBLE.	nil	nil	nil	nil	24	ORIF right parasymphysis left condyle	MAXIPLATE MINIPLATE	Yes	no call us	no callus	no callus	soft callus
23	1120080001	Mr.BALASUBRAMANI. S	35	M	RTA	15	unilateral mandible	unilateral zygoma	None	LEFT PARASYMPHYSIS	nil	nil	nil	nil	72	ORIF left parasymphysis right condyle right zygoma elevation	MAXIPLATE MINIPLATE	No	no call us	no callus	no callus	soft callus

S.No	ID No	NAME	AGE (YEARS)	SEX	MODE OF INJURY	GCS	CT FACE FINDINGS		Tooth	SITE OF MANDIBLE FRACTURE	ASSOCIATED INJURIES				INJURY TO SURGERY TIME (hrs)	PROCEDURE	TYPE OF FIXATION	POST OP IMF	ULTRASONOGRAM			
24	1120105463	Mr.RAJ	60	M	RTA	15	unilateral mandible	bilateral maxilla	None	RIGHT PARASYMPHYSIS, RIGHT SUBCONDYLAR PROCESS	nil	nil	nil	nil	10	ORIF right parasymphysis left zygoma	MAXIPLATE MINIPLATE	Yes	no callus	no callus	soft callus	hard callus
25	1111041977	Mr.SRINIVASAN	56	M	RTA	15	unilateral mandible	unilateral zygoma	None	RIGHT PARASYMPHYSIS	Yes	Nil	Nil	Yes	10	ORIF right parasymphysis	locking plate	No	no callus	no callus	soft callus	soft and hard callus
26	1120103011	Mr.GOKUL RAJ .D	21	M	RTA	15	unilateral mandible	unilateral maxilla	None	LEFT ANGLE OF MANDIBLE	Nil	Nil	Nil	Nil	160	ORIF - right parasymphysis	MINIPLATE	Yes	no callus	no callus seen	soft callus	hard callus
27	1120104617	Mr.MANI.M	38	M	RTA	15	bilateral mandible	bilateral maxilla	None	BILATERAL CONDYLAR HEAD, UNDISPLACED RIGHT PARASYMPHYSIS	nil	nil	nil	nil	60	Right parasymphysis	MINIPLATE	Yes	no callus	no callus	no callus	soft and hard callus
28	1120103532	Mr.SIVASHAKTHIBALUMAR	19	M	RTA	15	unilateral mandible	unilateral maxilla	Multiple avulsed teeth 11 21 22 42 41 31	RIGHT PARASYMPHYSIS	nil	nil	nil	nil	13	ORIF right parasymphysis of mandible	maxiplate. miniplate	Yes	no callus	no callus	soft callus	hard callus
29	1120112384	Mr.RAJENDRAN.V	28	M	RTA	15	unilateral mandible	unilateral maxilla	None	PARASYMPHYSIS	nil	nil	nil	Yes	90	ORIF right parasymphysis	MINIPLATE	No	no callus	no callus	no callus	soft callus seen
30	1120113730	Mr.BHASKARAN	33	M	RTA	15	bilateral mandible	unilateral zygoma	none	BILATERAL PARASYMPHYSIS OF MANDIBLE	nil	nil	nil	nil	24	ORIF bilateral parasymphysis	MAXIPLATE	no	no callus	soft callus seen	soft callus	hard callus
31	1120081980	Mr.RADHAKRISHNAN	29	M	RTA	15	bilateral mandible	bilateral maxilla	multiple upper anterior teeth	MANDIBULAR SYMPHYSIS AND LEFT CONDYLE	nil	nil	nil	nil	76	ORIF - symphysis mandible	MAXIPLATE	yes	no callus	no callus	no callus	soft callus
32	1120114252	Mrs.LINGAMMAL	65	F	RTA	15	bilateral mandible			BILATERAL CONDYLE AND LEFT PARASYMPHYSIS	yes	nil	nil	yes	12	ORIF - left parasymphysis and bilateral condyle	MINIPLATE	no	no callus	no callus	no callus	soft callus
33	1120115453	Mr.GNANAVADIVEL	40	M	RTA	15	communited unilateral mandible			SYMPHYSIS OF MANDIBLE.	nil	nil	nil	yes	76	ORIF - symphysis mandible	MAXIPLATE	yes	no callus	no callus	no callus	soft callus
34	1120051707	Mr.FAIZAL RAHMAN A	44	M	RTA		bilateral mandible	bilateral maxilla	loose teeth 21,22,23	FRACTURES OF BOTH MANDIBULAR CONDYLES - FRACTURE OF MANDIBULAR SYMPHYSIS AND	nil	nil	nil	nil	76	ORIF - left parasymphysis	MAXIPLATE	yes	no callus	no callus	no callus	soft callus

S.No	ID No	NAME	AGE (YEARS)	SEX	MODE OF INJURY	GCS	CT FACE FINDINGS		Tooth	SITE OF MANDIBLE FRACTURE	ASSOCIATED INJURIES				INJURY TO SURGERY TIME (hrs)	PROCEDURE	TYPE OF FIXATION	POST OP IMF	ULTRASONOGRAM			
										FRACTURE OF LEFT PARASYMPHYSEAL FRACTURE OF MANDIBULE												
35	1120123177	Mr.DIVAGAR.M	24	M	RTA	15	unilateral mandible	unilateral zygoma	NIL	FRACTURE LEFT BODY OF MANDIBLE	nil	nil	nil	nil	48	ORIF - Left body of mandible	MINIPLATE	yes	no call us	no callus	soft callus	hard callus
36	1120122647	Mr.SUDHEESH. G	25	M	RTA	15	bilateral mandible	bilateral maxilla	nil	RIGHT PARASYMPHYSIS AND LEFT SUB CONDYLE	nil	nil	nil	yes	46	ORIF - left symphysis and condyle	MAXIPLATE MINIPLATE	yes	no call us	no callus	soft callus	soft callus
37	1120124498	Mr.SUBBIAH	25	M	RTA	15	unilateral mandible	bilateral maxilla	avulsed 22, 32	LEFT PARASYMPHYSIS	nil	nil	nil	nil	40	ORIF - left parasymphysis and maxilla	MAXIPLATE MINIPLATE	yes	no call us	no callus	soft callus	soft and hard callus
38	1120120754	Mr.SIVA PRASAD. B	23	M	RTA	15	bilateral mandible		nil	SYMPHYSIS AND BILATERAL CONDYLE	nil	nil	nil	nil	192	ORIF - symphysis mandible	MAXIPLATE	yes	no call us	no callus	no callus	soft callus
39	1121010120	Mrs.CHITRA.E	46	FEMALE	machine	15	bilateral mandible	bilateral maxilla	nil	bilateral parasymphysis of mandible	nil	nil	nil	yes	48	ORIF - bilateral parasymphysis of mandible	MAXIPLATE	no	no call us	no callus	no callus	soft callus
40	1121011713	Mr.VIGNESHWARAN	25	MALE	RTA	15	bilateral mandible	bilateral maxilla	dentoalveolar fracture 41,42	right symphysis ,bilateral condyle	nil	nil	yes	yes	40	ORIF - symphysis mandible, bilateral condyle	maxiplate. miniplate	no	no call us	no callus	soft callus	hard callus
41	1121012560	Mr.MAHESHWARAN	38	MALE	RTA	15	bilateral mandible		nil	fracture of left body of mandible and right ramus of mandible	nil	nil	nil	yes	12	ORIF - ramus, left body	MINIPLATE	yes	no call us	no callus	no callus	soft callus
42	1121012963	Mr.TAMIL MANI	23	MALE	RTA	15	bilateral mandible		nil	Fracture of bilateral parasymphysis of mandible	nil	nil	nil	nil	46	ORIF - bilateral parasymphysis	MAXIPLATE	no	no call us	no callus	no callus	soft callus
43	1121015646	Mr.RAJKUMAR K	29	MALE	RTA	15	unilateral mandible		nil	fracture of left parasymphysis of mandible	nil	nil	nil	yes	45	ORIF- left parasymphysis	MAXIPLATE	no	no call us	no callus	soft callus	soft and hard callus
44	1121015636	Mr.PRITHIV RAJAN T	23	MALE	RTA	15	bilateral mandible	bilateral maxilla	nil	fracture of left parasymphysis and right condyle of mandible,	nil	nil	nil	nil	46	ORIF- left parasymphysis	MAXIPLATE	yes	no call us	no callus	soft callus	hard callus
45	1121015703	Mr.RAJKAPOOR R	19	MALE	RTA	15	bilateral mandible		nil	fracture of bilateral mandible - right parasymphysis and left angle of mandible	nil	nil	nil	nil	48	ORIF - right parasymphysis and left angle	MAXIPLATE MINIPLATE	no	no call us	no callus	no callus	soft callus

S.No	ID No	NAME	AGE (YEARS)	SEX	MODE OF INJURY	GCS	CT FACE FINDINGS		Tooth	SITE OF MANDIBLE FRACTURE	ASSOCIATED INJURIES				INJURY TO SURGERY TIME (hrs)	PROCEDURE	TYPE OF FIXATION	POST OP IMF	ULTRASONOGRAM			
46	1121015935	Mr.CHELLADURAI	32	MALE	RTA	15	unilateral mandible	unilateral zygoma	nil	left mandibular body fracture	nil	nil	nil	nil	47	ORIF - left parasymphysis	MINIPLATE	no	no callus	no callus	soft callus	soft and hard callus
47	1121016517	Mr.KOWSICK NIVAS	23	MALE	RTA	15	bilateral mandible		fracture teeth 11 21 22	fracture of left parasymphysis and bilateral condylar process of mandible	nil	nil	nil	nil	20	ORIF - left parasymphysis	MAXIPLATE MINIPLATE	yes	no callus	no callus	no callus	soft callus
48	1121021396	Mr.SAKTHIVEL	49	MALE	RTA	15	bilateral mandible		nil	fracture of left parasymphysis and right angle of mandible	nil	nil	nil	nil	12	ORIF - left parasymphysis	MINIPLATE	no	no callus	no callus	no callus	soft callus
49	1121021053	Mrs.CHINNAMMAL	70	FEMALE	assualt	15	unilateral mandible		mobile 32, 33	fracture of left parasymphysis of mandible	nil	nil	nil	nil	100	ORIF - left parasymphysis	MINIPLATE	no	no callus	no callus	no callus	soft callus
50	1121020124	Mr.KALAISELVAM	43	MALE	RTA	15	unilateral mandible		none	right angle of mandible	nil	nil	nil	yes	12	ORIF - angle of mandible	MINIPLATE	no	no callus	no callus	soft callus	soft and hard callus
51	1121020851	Mr.PICHAIMUTHU	60	MALE	RTA	15	unilateral mandible	bilateral maxilla	missing multiple upper and lower teeth	LEFT PARASYMPHYSIS OF MANDIBLE	yes	nil	nil	nil	47	ORIF - left parasymphysis	MAXIPLATE	yes	no callus	no callus	soft callus	soft and hard callus
52	1121021802	Mr.CHINNA DURAI	25	MALE	RTA	15	bilateral mandible	unilateral zygoma	avulsed 31, 32, 33, 34, 41	bilateral parasymphysis	nil	nil	nil	nil	14	ORIF - bilateral parasymphysis	MAXIPLATE	no	no callus	no callus	no callus	soft callus
53	1121022713	Mr.SEKAR. M	30	MALE	RTA	15	communited unilateral mandible		loss of lower anterior teeth 31, 32,33. 41,42,43	right parasymphysis	nil	nil	nil	nil	12	ORIF - right parasymphysis	MINIPLATE	no	no callus	no callus	no callus	soft callus
54	1121024005	Mr.SANTHOSH KUMAR A N	27	MALE	RTA	15	bilateral mandible	unilateral zygoma	none	left parasymphysis ,bilateral condyle of mandible	nil	nil	nil	nil	48	ORIF - left parasymphysis	MINIPLATE	no	no callus	no callus	no callus	soft callus
55	1121020124	Mr.KALAISELVAM	43	MALE	RTA	15	unilateral mandible		None	angle of mandible	nil	nil	nil	nil	40	ORIF - angle of mandible	MINIPLATE	no	no callus	no callus	soft callus	hard callus
